# Functional and Organic Diseases of the Stomach

**Published:** 1896-06

**Authors:** 


**Functional and Organic Diseases of the Stomach.** By Sidney Martin, M.D., F.R.S. Pp. xvi., 505. Edinburgh: Young J. Pentland. 1895.

So many years have elapsed since an authoritative treatise on diseases of the stomach has appeared from British sources, and so much advance has been made in recent years in our knowledge of the pathology of disorders of the stomach, that this book will be perused with great interest. The gain in knowledge of gastric affections has chiefly resulted from the systematic examination of the gastric contents at various stages after a meal and in various diseases. Although a certain part of this work has been done in England, and much of the rest is accessible through translations, yet the bulk remains in German and French books and journals which do not come in the way of the general reader. The first question that occurs to one's mind on taking up this book is, then, whether it gives a satisfactory account of the best of all this recent work. On the whole, this question can be answered in the affirmative. The reader will find sufficient information as to what has been done in this direction, and sufficient guidance to enable him to apply these modern methods in his own practice. This section of the book is succinct, and the matter judiciously selected. The author has evidently limited himself to the consideration of those methods that can be used in ordinary practice, without involving a large expenditure of time or great difficulty of application. For further information on more elaborate methods of procedure the reader is referred to the memoirs of the original workers. On questions such as the importance and significance in diagnosis of the presence in excess or absence of HCl from the gastric juice, the author takes well-founded and judicious views.

The first chapters, dealing with digestion in the stomach, the digestibility of various articles of diet, the points to be borne in mind in the selection of food-stuffs and arrangement of dietaries, leave little to be desired. The next chapters on the pathology of indigestion are also excellent, and contain a large amount of valuable information.

Seeing the confusion that has arisen from the number and variety of digestive disorders described, the author has made an attempt to divide functional diseases of the stomach into two groups, which he terms gastric irritation and gastric insufficiency respectively. He makes a further group—gastritis—to include those cases generally described as gastric catarrh. In gastric insufficiency there is diminution in the functional capacity of the organ without gross disease. The chapters on treatment of acute and chronic affections of the stomach are very good, especially with regard to the arrangement of the dietary in these cases. The last three chapters deal with dilatation, ulcer, and cancer of the stomach respectively. Of these, the one on ulcer is the best; that on dilatation does not deal sufficiently fully with the subject, perhaps partly because the author has incidentally considered minor degrees of dilatation in discussing gastric insufficiency and irritation. The chapter on cancer also hardly seems to us to enter thoroughly enough into all the aspects of this important disease.

Taking the book as a whole, it is much to be commended, and is especially good in the parts indicated above. We can recommend it to our readers as well worth reading, more particularly with regard to modern methods of diagnosis and treatment in gastric affections. The book is printed in large and clear type, and the work turned out in this publisher's usual excellent style.

